# Alpha and Beta Corticomotor Phase Dynamics Shape Visuomotor Control on a Single-Trial Basis

**DOI:** 10.1523/JNEUROSCI.0765-25.2025

**Published:** 2026-01-20

**Authors:** Alice Tomassini, Francesco Torricelli, Luciano Fadiga, Alessandro D’Ausilio

**Affiliations:** ^1^Dipartimento di Neuroscienze e Riabilitazione, Università degli Studi di Ferrara, Ferrara 44121, Italy; ^2^Center for Translational Neurophysiology of Speech and Communication, Italian Institute of Technology, Ferrara 44121, Italy

**Keywords:** alpha, beta, corticomotor coherence, feedback-based control, neuronal oscillations, visuomotor processing

## Abstract

A central question in sensorimotor neuroscience is how sensory inputs are mapped onto motor outputs to enable swift and accurate responses, even in the face of unexpected environmental changes. Here, we leverage corticomotor coherence as a window into the dynamics of sensorimotor loops and explore how it relates to online visuomotor control. We recorded brain activity using electroencephalography, while human participants (of either sex) performed an isometric tracking task involving transient, unpredictable visual perturbations. Our results show that coherence between cortical activity and motor output (force) in the alpha band (8–13 Hz) is associated with faster motor responses, while beta-band coherence (18–30 Hz) promotes more accurate control, which is in turn linked to a higher likelihood of obtaining rewards. Both effects are most pronounced near the onset of the perturbation, underscoring the predictive value of corticomotor coherence for sensorimotor performance. Single-trial analyses further reveal that deviations from the preferred corticomotor phase relationship are associated with longer reaction times and larger errors, and these phase effects are independent of power effects. Thus, beta-band coherence may reflect a cautious, reward-efficient control strategy, while alpha-band coherence enables quicker, though not necessarily efficient, motor responses, indicating a complementary, reactive control mode. These results highlight the finely tuned nature of sensorimotor control, where different aspects of sensory-to-motor transformations are governed by frequency-specific neural synchronization on a moment-to-moment basis. By linking neural dynamics to motor output, this study sheds light on the spectrotemporal organization of sensorimotor networks and their distinct contribution to goal-directed behavior.

## Significance Statement

How the brain integrates sensory information with ongoing motor plans to enable quick and accurate responses to unpredictable events remains unclear. By analyzing the oscillatory coupling between brain activity and motor output (force), we identify patterns that selectively govern key attributes of effective behavior. Oscillatory coupling in the alpha band (∼10 Hz) supports rapid reactions, while coupling in the beta band (∼25 Hz) promotes cautious, reward-driven control. These findings enhance our understanding of how the brain organizes sensorimotor processes, allowing us to flexibly adapt to changing environments and goals. This research has potential implications for developing more effective treatments for motor disorders, improving human–machine interactions, and advancing robotic control systems.

## Introduction

To maximize rewards or avoid potentially harmful failures, motor behavior must be conveniently fast and accurate, even in the face of unpredictable events. Achieving this level of performance requires integrating sensory and motor signals across various spatiotemporal scales, a process that is made possible by the highly distributed anatomofunctional architecture of the sensorimotor system (Scott, 2016; Bizzi and Ajemian, 2020; Cruz et al., 2023). The coordinated activity of these multilevel control loops is likely governed by frequency-specific oscillatory dynamics, which reflect functional interactions between relevant neuronal populations along sensorimotor pathways, extending to the periphery where motor outputs are generated (Fries, 2005; Buzsáki, 2009; Siegel et al., 2012).

Evidence suggests that the motor output retains traces of this multiscale activity (MacKay, 1997; Oya et al, 2020; Tomassini et al., 2020; Emanuele et al., 2024). Measuring oscillatory phase synchronization, i.e., coherence, between the brain and the periphery—such as muscle activity, force, or kinematics—can thus provide insights into the dynamics of sensorimotor loops. Indeed, corticomotor coherence likely reflects both efferent drive and re-afferent signaling, indexing the operations occurring within a somatosensory-motor loop (Baker, 2007; Witham et al., 2011; Aumann and Prut, 2015; Oya et al., 2020). This dual nature—motor and (somato)sensory—has traditionally been ascribed to the coherence observed in the beta band (13–30 Hz), aligning with the prevailing view of beta activity as being involved in monitoring and maintaining the current proprioceptive-motor state, often at the expense of movement initiation (Gilbertson et al., 2005; Kristeva et al., 2007; Pogosyan et al., 2009; Engel and Fries, 2010). The beta rhythm is considered the main oscillatory mode of the motor system (Jasper and Penfield, 1949; Murthy and Fetz, 1992) and typically stands out as the strongest component of the coherence spectrum, particularly during sustained tasks (Baker et al., 1997). However, corticomotor coherence is a spectrally heterogeneous phenomenon, comprising distinct components, such as the alpha band (8–13 Hz), which exhibit a similar scalp distribution contralateral to the effector yet may serve different functional roles (Mima and Hallett, 1999; Raethjen et al., 2002; Mehta et al., 2014; Tomassini et al., 2020).

Previous studies have primarily focused on linking corticomotor coherence to basic motor parameters (Brown et al., 1998; Kilner et al., 2000) and performance (Kilner et al., 1999; Kristeva et al., 2007; Omlor et al., 2011; Suzuki and Ushiyama, 2020; Mongold et al., 2022). However, a recent study has demonstrated that corticomotor coherence in the alpha band is also relevant to perceptual performance (Tomassini et al., 2020). Specifically, increases in coherence within this band may correspond to heightened visual excitability, as evidenced by the increased detection rates for unpredictable, near-threshold stimuli. This bolsters the view that corticomotor coherence is not solely motor-related but is also fundamentally tied to sensory—including visual—processing, with behavioral implications extending to perception.

Collectively, the evidence suggests that processing at the sensory (input) and motor (output) stages may be intrinsically coupled (Tomassini et al., 2017; Benedetto et al, 2020). Our central hypothesis is that this coupling plays a pivotal role in enabling efficient closed-loop control. To test this, we investigated whether ongoing corticomotor coherence shapes closed-loop visuomotor performance and whether spectrally specific phase dynamics in the alpha and beta bands differentially govern key behavioral outcomes—specifically, speed and accuracy.

To this end, we simultaneously recorded motor output (force) and brain activity using electroencephalography (EEG), while human participants engaged in a continuous isometric visuomotor tracking task that included transient, unpredictable visual perturbations. Participants were instructed to counter these perturbations as quickly and accurately as possible. Performance was incentivized through rewards, which were contingent on meeting a fixed accuracy criterion within an individually titrated time window. We then examined both coherence and the single-trial corticomotor phase relationship prior to the perturbation and evaluated their predictive value for subsequent online visuomotor control, as indexed by the speed and accuracy of perturbation compensation.

## Materials and Methods

### Participants

Thirty-six healthy participants were recruited for the study. They were all naive with respect to the aims of the study and were paid (25 €) for their participation. Participants were right-handed (by self-report) and had normal or corrected-to-normal vision. The study and experimental procedures were approved by the local ethics committee (Comitato Etico di Area Vasta Emilia Centro, ref: EM255–2020_UniFe/170 592). Participants provided written informed consent after receiving explanations of the experimental task and procedures in accordance with the guidelines of the local ethics committee and the Declaration of Helsinki. Data from six participants were excluded from the analysis due to excessive noise in the EEG signal (*n* = 3), noncompletion of the experiment (*n* = 2), and technical issues with data acquisition (*n* = 1). The analysis was then performed on the data from the remaining 30 participants (17 females; age, 25.1 ± 3.7 years, mean ± SD).

### Experimental design

**Figure 1.**
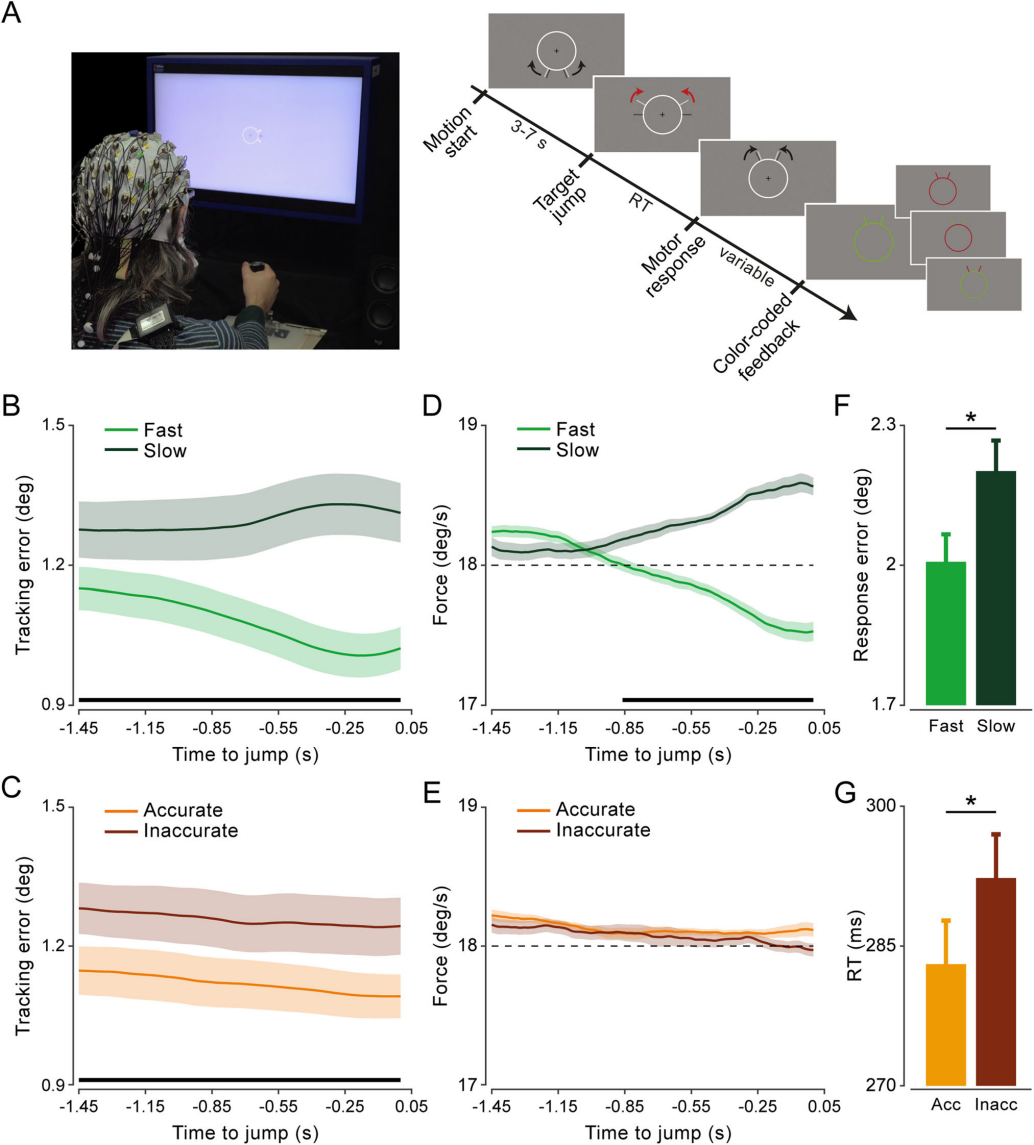
Experimental design and behavioral performance. ***A***, Left, EEG (64-channel) and force were recorded while human participants applied force with their right hand on an isometric joystick to control the speed of a cursor (dark gray bars) and track a target (light gray bars) moving at a constant angular velocity (18°/s) along a circular path. Right, Timeline of “jump” trials: after a random time from the start of its motion, the target jumped forward and participants were required to realign the cursor to the target as quickly and accurately as possible. At the end of each trial, participants received color-coded feedback on their performance (the schematic does not exactly represent the visual display shown in the actual experiment, as the stimulus size is enlarged and the black and red arrows are included for illustrative purposes only; see Materials and Methods for further details). ***B***, Tracking error (absolute difference between the angular positions of the cursor and target) as a function of time to target jump (time zero) for fast and slow trials (based on a median split). ***C***, Same as in ***B*** but for accurate and inaccurate trials (based on a median split). ***D***, Force, encoding cursor speed (degrees/second), as a function of time to target jump for fast and slow trials. ***E***, Same as in ***D*** but for accurate and inaccurate trials. Shaded areas represent ±1 SEM. Black horizontal lines indicate the time points that survive paired *t* test statistics (two-tailed) for the relevant contrast (fast vs slow, accurate vs inaccurate) with FDR correction for multiple comparisons across time. ***F***, Response error for fast and slow trials. ***G***, RT for accurate and inaccurate trials. Error bars indicate ±1 SEM. Reported statistical significance is based on two-tailed paired *t* tests.

Participants sat in a dark room in front of a screen (24 in, 1,920 × 1,080 pixels, 120 Hz; VIEWPixx/EEG, VPixx Technologies) at a viewing distance of ∼60 cm. With their right hand, they held a custom-made isometric joystick which was securely fixed to a rigid support (Fig. 1*A*, left). The joystick was connected to a six-axis force/torque sensor (Gamma F/T transducer, ATI Industrial Automation), which enabled continuous hand force measurements. The analog output of the force/torque transducer was recorded with the EEG system and also acquired continuously with another data acquisition board (MCC-USB1608G, 5,000 Hz; Digilent), allowing (pseudo) real-time control of the visual display. The visual display and acquisition of force/torque data were controlled using MATLAB (R2021b; The MathWorks) and Psychtoolbox-3 (Brainard, 1997).

Participants performed an isometric visuomotor tracking task. They exerted a constant level of force (target force; see below) by pulling the joystick through wrist abduction, primarily involving contraction of the extensor carpi radialis longus (ECRL) muscle. The force exerted (along one main sensor axis, here torque along the *x*-axis) was used to control the angular velocity of a cursor and track a target moving at a constant speed along a circular path. Both the cursor and target consisted of two small mirror-image-arranged bars (dark and light gray, respectively; same size, 0.1 × 1° visual angle; Fig. 1*A*, right). The force output was converted into the angular velocity of the cursor, *ω_crsr_*, using the following formula:

$$\omega_{crsr}(t)\;=\;\omega_{tgt}\;*\;\frac{F(t)}{F_{tgt}},\;(1)$$

where *ω_tgt_* is the constant angular velocity of the target, *F_tgt_* is the target force, and *F*(*t*) is the force output at time *t*. Finally, the cursor angular position $\theta_{crsr}$ was derived as follows:

$$\theta_{crsr}(t+\Delta t)\;=\;\theta_{crsr}(t)\;+\;\Delta t\;\omega_{crsr}(t+\Delta t),\;(2)$$

where Δ*t* is the interframe interval (∼8.3 ms; frame rate, 120 Hz). Notably, the cursor position could only be changed in the forward direction, that is, in the same direction as the motion of the target (i.e., the cursor could not move backward).

Each trial began with the appearance of the fixation cross (black; 0.3 × 0.3°), circular path (white; radius, 3.3°; width, 0.2°), and target on a medium-gray background. The fixation cross and circular path were displayed at the center of the screen, whereas the target appeared randomly in one out of four positions along the path (0, 90, 180, or 270°). After a variable time (drawn at random from a uniform distribution between 0.6 and 1.8 s), the cursor was shown at the same position as the target, and the target began to move at a constant speed (18°/s). Participants had to apply force to track the target as accurately as possible, that is, to keep the cursor spatially aligned with the target. Therefore, the ideal performance was to quickly catch up with the target at the start of the trial and then keep the force exerted as close to *F*_tgt_ as possible.

In most trials (90%, jump trials), the target was briefly accelerated at an unpredictable time, drawn randomly from a uniform distribution between 3 and 7 s after the start of the target motion. Therefore, the target jumped forward in the same direction as its motion (target displacement, 18° in 0.2 s). Participants had to compensate for the target jump by realigning the cursor with the target as quickly and accurately as possible (see below). In the remaining trials (10%, catch trials), the target completed its trajectory at a constant speed, and participants had to continue tracking until the target had traveled the entire path (180°, corresponding to a total duration of 10 s).

At the end of each trial, participants received color-coded feedback on both tracking performance and, in jump trials only, the compensatory response to the target jump. Specifically, tracking performance was assessed by computing the tracking error, defined as the absolute difference between the angular positions of the cursor and the target—calculated over the prejump period for jump trials and over the entire tracking period for catch trials (excluding the first 0.5 s following target motion onset). Participants received positive feedback—indicated by the circular path turning green—if their tracking error during the relevant interval remained below a predefined threshold, i.e., the criterion error of 4.6°; if the error exceeded this criterion, negative feedback—circular path turning red—was provided instead. Regarding the response to the jump, participants were given a limited time interval—the criterion time (or deadline)—to realign the cursor to the target after its jump; the criterion time was set at the beginning of the experiment equal to 0.6 s (except for Participant 1, for whom it was equal to 0.4 s) and then slightly adapted based on performance (see below). Participants received positive/negative feedback—indicated by the target turning green/red—if the error (again the absolute cursor-target deviation in angular position) was below/above the criterion error (4.6°) for 1 s after passing the criterion time. After this 1°s interval, the trial ended. The criterion time was adjusted on a block-wise basis according to individual performance. Specifically, it was decreased by 50 ms if the reward rate in the previous block (60 trials; see below) exceeded 80%; increased by 50 ms if it fell between 40 and 60%; increased by 100 ms if it was below 40%; and left unchanged if it ranged between 60 and 80%.

After a preliminary phase for task familiarization, participants performed six blocks of 60 trials each (except for Participant 1, who performed seven blocks of 50 trials each). The type of trial (jump/catch) and initial target position were fully balanced and randomized within each block of trials.

### Data recording

EEG data were recorded continuously (sampling rate, 1,000 Hz) during the experiment using a 64-channel active electrode system (Brain Products). Electrooculograms were recorded using four electrodes from the cap (FT9, FT10, PO9, and PO10) placed at the bilateral outer canthi and below and above the right eye to record horizontal and vertical eye movements, respectively. An electrode placed on the left mastoid was used as the online reference. The impedance of the electrodes was kept below 15 kΩ.

Additionally, electromyographic (EMG) data from a right arm muscle in the radial deviation of the wrist (ECRL) were recorded using a belly–tendon montage, amplified (50,000×), and bandpass filtered (10–500 Hz) with a D360 amplifier (Digitimer). The muscle of interest was identified via standard palpation procedures. EMG data were also acquired with the EEG amplifier (1,000 Hz).

All the recorded signals (EEG, EMG, and force) were synchronized with the visual display using the built-in VIEWPixx TTL triggering system.

### Data analysis

Data analysis was performed with MATLAB using custom-made code, the FieldTrip toolbox (Oostenveld et al., 2011; http://www.fieldtriptoolbox.org; RRID:SCR_004849), and the SleepTrip extension (http://www.sleeptrip.org; RRID:SCR_017318).

#### Behavioral analysis

Behavioral performance was assessed both in terms of continuous tracking (tracking error) and in response to the perturbation [response error and reaction time (RT)].

Tracking error was quantified as the absolute difference between the angular positions of the cursor and the target, calculated over a prejump window from −1.5 to 0 s relative to the target jump in jump trials (Fig. 1*B*,*C*) and from 2 to 8 s after target motion onset in catch trials.

Performance in response to the target jump was evaluated by calculating two main metrics: (1) response error and (2) reaction time (RT). Response error was calculated in the same way as the tracking error (i.e., absolute difference between the angular positions of the cursor and the target) but averaged over the final 1 s window. This corresponds to the time period during which the error had to remain below the criterion (4.6°) for participants to receive positive feedback (see above, Experimental design). To estimate the RT, the force signal (along the sensor axis where the force was mainly exerted, i.e., the *x*-axis torque) was first low-pass filtered (30 Hz; Butterworth, two-pass, sixth order). Response onset was determined as the first sample of a consecutive series of 50 samples (0.05 s), starting with 75 samples (0.075 s) after the target jump, where the first derivative of the force exceeded 5% of its maximum value. The RTs thus calculated were then checked on a trial-by-trial basis and manually corrected if necessary (<3% of the trials). In some participants (73%) and a small percentage of trials (1.96 ± 1.17%; mean ± SD), response onset could not be reliably determined; these trials were excluded from all the analyses involving postjump performance.

In addition, a composite performance score—“efficiency”—was computed on a trial-by-trial basis to capture how closely behavior in response to the target jump was steered toward reward attainment, using the following formula:

$$\mathrm{efficiency}=\;\mathrm{response}\;\mathrm{erro}r_{\mathrm{norm}}\times RT_{\mathrm{norm}},\;(3)$$

where $\mathrm{response}\;\mathrm{erro}r_{\mathrm{norm}}$ and $RT_{\mathrm{norm}}$ correspond to the response error and RT, respectively, normalized by their associated performance criteria used during the experiment, namely, the fixed criterion error (4.6°) and the variable criterion time (deadline). Values of 1 or lower for both component terms indicate that performance met or outperformed the respective criteria. Notably, rewards—signaled via green-colored feedback at the end of the trial—were delivered when the criterion error was met throughout the 1 s window following the deadline (see also above, Experimental design). Therefore, the efficiency score provides an integrated estimate of how successfully participants balanced speed and accuracy to achieve the task goal and obtain reward.

#### EEG preprocessing

Continuous EEG data were first bandpass filtered (0.1–300 Hz; Butterworth, two-pass, fourth order) and then segmented into epochs of varying length extending from 1 s after the start of the target motion until either the jump of the target (jump trials) or 1 s before the end of the trial (catch trials). The segmented data were then visually checked for bad channels and artifacts in the time domain. Independent component analysis (ICA; implemented in FieldTrip using the “runica” method) was used to identify and remove residual artifacts related to eye movements and cardiac activity. Components were evaluated based on their spatial topographies, temporal dynamics, and, when necessary, spectral characteristics. Noisy EEG channels were excluded from the ICA analysis and subsequently interpolated using a distance-weighted nearest–neighbor approach.

#### Spectral analysis and coherence

Fourier-based analysis (frequency range, 5–35 Hz) of the force was performed on Hanning-tapered 1.5 s data windows (nonoverlapping, zero-padded to 2 s), extracted from continuous tracking periods (jump trials, from −1.5 to 0 s relative to the target jump; catch trials, from 2 to 8 s after target motion onset). To estimate the periodic components, the resulting power spectrum was parameterized using FOOOF (as implemented in the SleepTrip toolbox; settings, aperiodic mode, “fixed”; max peaks, 4; peak width, 0.5 −12 Hz; min peak height, 2 dB; peak threshold, 2 SD; proximity threshold, 1 SD; Donoghue et al., 2020). Individual alpha- and beta-band periodic components were identified as the center frequencies of the fitted peaks that fell in the 5–15 Hz and 15–35 Hz range, respectively (the peak with higher power was considered if >1 peak met this criterion).

To compute cortico-force phase coherence, we first applied short-time Fourier transform (frequency range, 5–35 Hz) on Hanning-tapered 0.3 s windows (overlapped by 50%, zero-padded to 2 s) extracted from the same data epochs (for both jump and catch trials) used for the spectral analysis (see above). We then computed the cross-spectral density (CSD) between the EEG (*i*) and force (*j*) signal Fourier spectra (*F*; ´denotes the conjugate) for each data window (*t*) and frequency (*f*) as follows:

$$\mathrm{CS}D_{ijt}(f)=F_{it}(f)\times F_{\;jt}{\left(f\right)}^{\prime }.\;(4)$$

Cortico-force coherence (COH) was then computed by dividing the length of the CSD vector average (over data windows) by the square root of the product of the average Fourier-based power estimates (*P*) of both signals (EEG and force), as follows:

$$\mathrm{CO}H_{ij}(f)=\frac{\left|\sum_{t=1}^{t}\;\mathrm{CS}D_{ijt(f)}\right|}{\sqrt{\left(\sum_{t=1}^{t}\;P_{it(f)}\right)\;*\;\left(\sum_{t=1}^{t}\;P_{\;jt(f)}\right)}}.\;(5)$$

Based on findings from a previous study (Tomassini et al., 2020) reporting distinct corticomotor coherence selectivity in the alpha and beta frequency ranges as a function of the lag between cortical and force signals, we repeated the coherence analysis by systematically shifting the EEG signals backward (negative lags) and forward (positive lags) in time relative to the force signals, ranging from −0.3 to +0.2 s in 0.025 s increments.

To test whether prejump coherence changed depending on reactive performance, we computed time-resolved estimates of lagged coherence separately for trials showing either short or long RTs (fast–slow trials), and small or large response errors (accurate–inaccurate trials) based on (separate) median splits of the data (jump trials only). Specifically, coherence was computed on 0.3 s sliding windows that were advanced from −1.45 to −0.25 s (step, 0.025 s) relative to the target jump. This analysis was repeated for lags from −0.3 to +0.1 s (step, 0.025 s). The median-split analysis was also repeated using the efficiency score (Eq. 3) to clarify the functional role of corticomotor coherence in relation to reward-oriented behavior.

#### Single-trial analysis

In addition to the classical coherence metric, we derived a point-by-point estimation of the phase synchronization strength, hereafter called “instantaneous coupling” (IC), using the same approach as that used by Rohenkohl et al. (2018). The IC quantifies for each trial and time point how close the phase relationship between the relevant signals (in this case, EEG and force) is to their “preferred” (mean) phase relationship. To this end, we first computed the CSD at each time point (*t*) and frequency (*f*; for each trial), using the formula previously described (Eq. 4).

The normalized average of the CSD across time (and trials) provides an estimate of the mean phase relation (the “preferred” phase) as follows:

$$\rho_{ij}(f)=\;\frac{\sum_{t=1}^{t}\;\mathrm{CS}D_{ijt(f)}}{\left|\sum_{t=1}^{t}\;\mathrm{CS}D_{ijt(f)}\right|}.\;(6)$$

We then computed the instantaneous deviation from the mean phase relation at each time point (and for each trial) as the “rotated CSD” as follows:

$${\mathrm{CSD}}_{ijt}^{\mathrm{rot}}(f)=\;\mathrm{CS}D_{ijt}(f)\;*\;\rho_{ij}{\left(f\right)}^{\prime }.\;(7)$$

By taking the cosine (cos) of the resulting phase angle (arg), we finally obtained the single-trial IC estimates, whose values range from −1 (observed phase relationship opposite to mean phase relationship) to 1 (observed phase relationship equal to mean phase relationship) as follows:

$$IC_{ijt}(f)=\cos\;(\arg({\mathrm{CSD}}_{ijt}^{\mathrm{rot}}(f))).\;(8)$$

This analysis employed the same Fourier-based estimates used in the computation of time-resolved coherence (see above) but focused specifically on the alpha (8–13 Hz) and beta (18–30 Hz) frequency bands. The analysis was also restricted to the lag and time windows identified in the previous median-split analysis (see above). Specifically, IC was averaged over the same time window (−0.35 to −0.3 s) for both the alpha and beta bands and over lag windows from −0.175 to −0.125 s for alpha and −0.025 to +0.025 s for beta, where the respective coherence modulations were centered.

#### Data stratification

To rule out that differences in corticomotor coherence between trial categories (fast vs slow; accurate vs inaccurate) were due to prejump differences in behavioral performance or oscillatory power, we employed a data stratification approach. Separate stratifications were conducted to match, as much as possible, the distributions of (1) tracking error, (2) force, and (3) oscillatory power across the relevant trial categories (fast vs slow; accurate vs inaccurate). Specifically, the relevant control variable was averaged in the 0.5 s before target jump. The distributions of the control variable for the two trial subsets were then divided into 20 equally spaced bins. The number of trials in each bin for the two subsets (fast vs slow; accurate vs inaccurate) was then equalized using a random subsampling procedure (as implemented in FieldTrip, function, ft_stratify; method, “histogram,” “equalbinavg”). For the stratification on oscillatory power, we used power in the alpha (8–13 Hz) and beta (18–30 Hz) band for the fast versus slow and accurate versus inaccurate comparisons, respectively. In both cases, power was averaged across the same selected electrodes as used in the statistical test of coherence (see below).

A similar stratification analysis was conducted to equalize the trial categories defined by one performance metric with respect to the other—that is, matching fast and slow trials on response error and accurate and inaccurate trials on RT.

#### Statistical analysis

No power analysis was used to decide on the sample size (i.e., the number of subjects). Sample size estimation was based on previous studies investigating corticomotor coherence (Bourguignon et al., 2017; Tomassini et al., 2020).

Statistical comparisons were conducted between trial categories defined by median splits based on either RT (fast vs slow), response error (accurate vs inaccurate), and efficiency (efficient vs inefficient). For comparisons of behavioral performance (Fig. 1*B–G*), we applied conventional paired sample *t* tests and, when appropriate, corrected for multiple comparisons across time using false discovery rate (FDR) control, as described by Benjamini and Yekutieli (2001). For comparisons of cortico-force coherence, we averaged coherence estimates across a selected group of electrodes (FC1, FC3, FC5, C1, C3, C5, CP1, CP3, and CP5). We then applied two-tailed cluster–based permutation tests (Maris and Oostenveld, 2007), which allow more effective handling of multiple comparisons in the case of multidimensional data (i.e., frequency, lag, and time). This nonparametric statistical approach consists of first selecting all samples exceeding an a priori decided threshold (uncorrected *p* < 0.05, two-tailed) for univariate statistical testing (dependent-sample *t* test) and then clustering them based on their contiguity along the relevant (frequency/lag/time) dimension(s). Cluster-level statistics were computed by taking the sum of the *t* values in each cluster. This sum is then used as a test statistic and evaluated against a surrogate distribution of maximum cluster *t* values obtained after permuting data across conditions (at the level of participant-specific condition averages). Surrogate distributions were generated using 10,000 permutations. The *p* value is given by the proportion of random permutations that yield a larger test statistic compared with that computed for the original data.

Statistical comparisons on stratified data (see above) were performed using one-tailed permutation tests, focusing on the alpha (8–13 Hz) and beta (18–30 Hz) bands for the fast versus slow and accurate versus inaccurate contrast, respectively. Statistical tests were restricted to specific lag intervals (alpha, −0.175 to −0.125 s; beta, −0.025 to +0.025 s) and a time window (−0.5 to −0.25 s) where significant coherence modulations had been identified in the main analyses based on median splits (see above).

In addition to statistical comparisons based on median splits, we performed linear mixed-effects (LME) model analysis. In this analysis, a single model was fitted to the data from all participants, incorporating fixed-effect factors (independent variables of interest or predictors) and random-effect intercepts (with participant as a grouping variable). The model behind this analysis can be written as follows:

$$Y_{ij}\;=\;\beta 0+u_{i}0+\beta n(\mathrm{predicto}r_{n_{ij}})+e_{ij},\;(9)$$

where *Y_ij_* denotes the response variable (i.e., RT or response error) for participant *i* (with *i* = 1, 2…30) and trial *j* (with *j* = 1, 2…total number of trials), *β*_0_ is the common intercept term, $u_{i}0$ is the random effect for the intercept (for each level of the grouping variable, i.e., for each participant *i*), *β_n_* is the fixed-effect term for predictor *n* (with *n* = 1, 2…total number of predictors), and *e_ij_* is the residual. Additionally, we conducted the same LME model analysis using a permutation approach, randomly shuffling the response variable (i.e., RT or response error) over 10,000 iterations. This allowed us to derive surrogate-based statistical thresholds for each predictor, defined as the 95th percentile (one-tailed test) of the resulting distribution of beta coefficients.

## Results

### Behavioral performance

Participants were able to track the target with the required accuracy in 72.4 ± 2.34% of the trials (and thus receive positive feedback), with an average tracking error of 1.19 ± 0.05° (mean ± SE). In jump trials, they responded with a transient increase in force 290 ± 50 ms after the target jump and eventually realigned the cursor to the target with an error of 2.1 ± 0.06°, successfully obtaining the reward (positive feedback) on their compensatory response in 59.3 ± 2.06% of the trials (mean ± SE).

Participants’ capacity to compensate for the target jump was systematically associated with their prejump tracking performance. Performance prior to the target jump (−1.45 to 0 s) was more accurate—that is, tracking errors were lower—in trials where participants also responded more quickly (shorter RTs) or more accurately (smaller response errors) to the target jump, compared with trials with slower or less accurate responses (comparisons based on median splits; all *p* < 0.001; two-tailed paired–sample *t* tests, with FDR correction across time; Fig. 1*B*,*C*). The ongoing force output—encoding cursor speed—also predicted participants’ readiness to counter the target jump, with faster trials exhibiting lower force than slower trials in the prejump period (from −0.85 to 0 s; all *p* < 0.04, FDR-corrected; Fig. 1*D*). However, force output did not predict response error (all *p* > 0.08, uncorrected; Fig. 1*E*).

This pattern of results was confirmed by separate LME model analysis on RT and response error (as dependent variables), using both the tracking error and force averaged from −0.85 to 0 s before the target jump as predictors. RT was significantly predicted by both prejump tracking error (*t*_(9377)_ = 20.2472; *p* < 0.0001; standardized beta coefficient = 0.1926) and force (*t*_(9377)_ = 22.3403; *p* < 0.0001; standardized beta = 0.2049), whereas response error was significantly predicted by tracking error (*t*_(9377)_ = 11.1298; *p* < 0.0001; standardized beta = 0.1150), but not by force (*t*_(9377)_ = –0.8418; *p* = 0.399; standardized beta = −0.0084).

In addition to their systematic association with prejump performance, the two performance metrics—RT and response error—also covaried: fast trials tended to exhibit smaller response errors and, thus, greater accuracy (*t*_(29)_ = –5.95; *p* < 0.0001; two-tailed paired–sample *t* test; Fig. 1*F*), while accurate trials, conversely, showed shorter RTs (*t*_(29)_ = –6.29; *p* < 0.0001; two-tailed paired–sample *t* test; Fig. 1*G*).

### Spectral and lag selectivity of corticomotor coherence

We first aimed to characterize corticomotor dynamics during continuous visuomotor control by examining the oscillatory coupling between EEG activity and force output.

**Figure 2.**
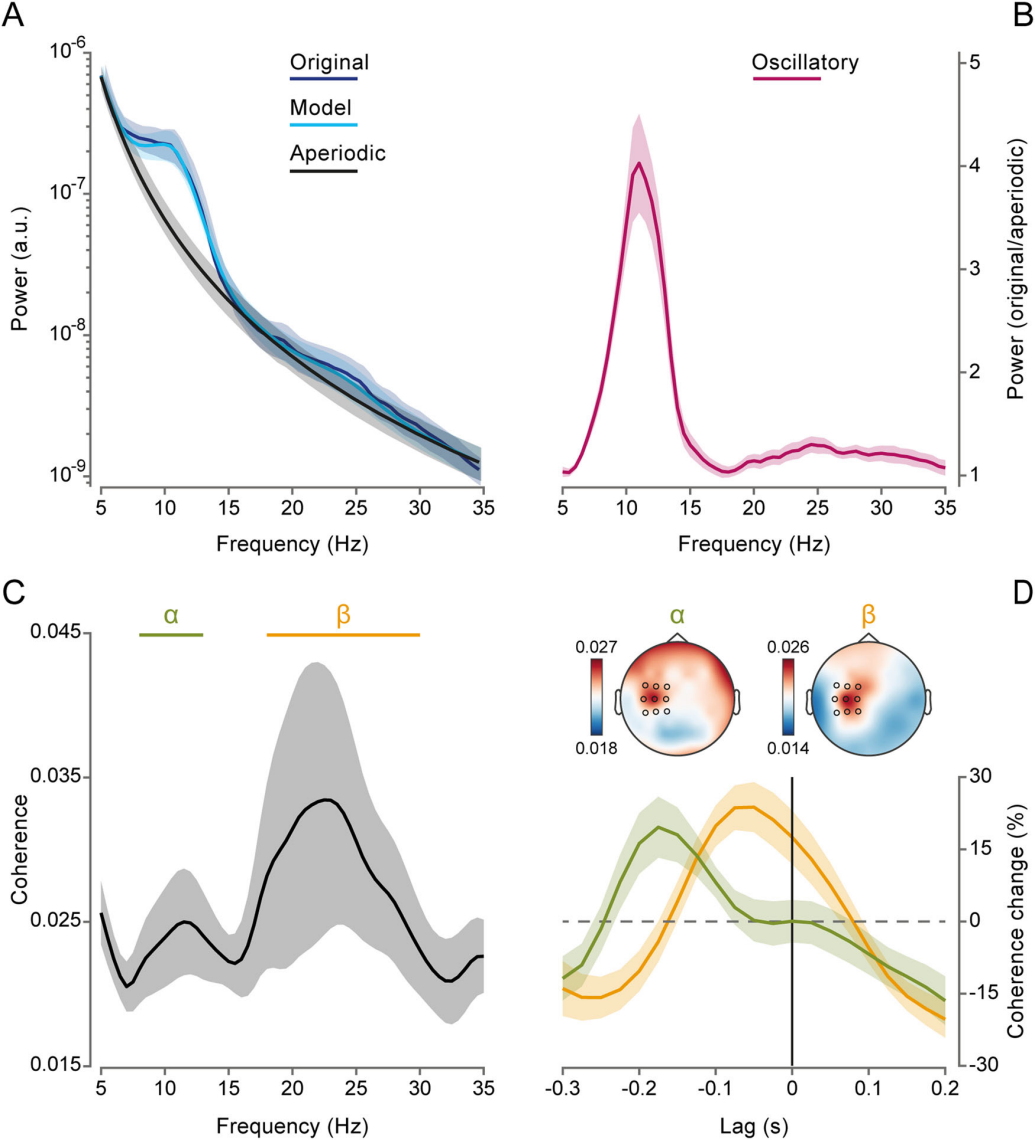
Spectral content of motor output (force) and corticomotor coherence. ***A***, Power spectrum of the force (blue) derived from Fourier-based analysis on Hanning-tapered 1.5 s data windows (nonoverlapping, zero-padded to 2 s) belonging to continuous tracking periods (for both “jump” and “catch” trials). The power spectrum was parameterized using FOOOF (Donoghue et al., 2020; full model fit, cyan), yielding an estimate of the aperiodic (1/*f*) component (gray). ***B***, Force oscillatory component plotted as the ratio between the force power spectrum and the estimated aperiodic component. ***C***, Corticomotor coherence spectrum at zero lag (no temporal shift between EEG and force signals), averaged over selected contralateral electrodes (FC1, FC3, FC5, C1, C3, C5, and CP1, CP3, CP5; highlighted in black in the topographic maps shown in panel ***D***). ***D***, Lag-tuning profiles of corticomotor coherence expressed as the percentage change in coherence relative to mean coherence across the entire range of lags (from −0.3 to +0.2 s), averaged over frequencies between 8 and 13 Hz (alpha, green) and between 18 and 30 Hz (beta, yellow); topographies are shown for alpha and beta coherence, averaged over lags (−0.3 to +0.2 s). Shaded areas represent ±1 SEM.

It is commonly reported that the force expressed under isometric conditions exhibits a distinctive spectral content featuring an alpha-band rhythm, often referred to as physiological tremor (McAuley and Marsden, 2000). Indeed, the force signal (during continuous tracking) showed a consistent periodic component (separable from the 1/*f* component; Fig. 2*A*,*B*), with oscillatory peaks in the alpha band observable in all participants (range, 8–13 Hz). Smaller amplitude peaks were also detectable in the beta band (range, 21–31 Hz), although in a much smaller percentage of participants (27%; see Fig. S1 for detailed single-participant results).

We analyzed the relationship between these peripheral rhythms and cortical activity by computing coherence during continuous tracking (i.e., excluding postjump data). Two distinct peaks were observed in the coherence spectrum, one in the alpha range (∼8–13 Hz) and the other in the beta range (∼18–30 Hz), which is in line with previous evidence (Tomassini et al., 2020; Fig. 2*C*; see Fig. S2 for additional spectral characterization and single-participant results). A recent study (Tomassini et al., 2020) demonstrated that cortico-force coherence in the alpha band has a distinctive lag-tuning profile, with coherence being maximized if an anticipatory time lag of cortical signals with respect to peripheral signals is taken into account. Therefore, we repeated the coherence analysis as a function of lag by systematically shifting cortical signals either backward (negative lags) or forward (positive lags) in time relative to the force signals. As shown in Figure 2*D*, the lag-tuning profile peaks at a more negative value for alpha coherence (lag, −0.175 s) compared with beta coherence (lag, −0.05 s), highlighting a pronounced lead of cortical alpha over peripheral alpha, in line with previously reported results (Tomassini et al., 2020).

The topographical distribution of coherence in both frequency bands closely resembles patterns commonly reported in other studies (Salenius et al., 1997; Schoffelen et al., 2005), with coherence predominantly concentrated on frontocentral electrodes contralateral to the hand effector (highlighted as black circles in the topographic maps in Fig. 2*D*). Subsequent analyses were performed by averaging coherence estimates across this relevant set of electrodes (i.e., FC1, FC3, FC5, C1, C3, C5, CP1, CP3, and CP5; see Fig. S3 for additional results on spectral parameterization of EEG signals at these sites).

### Alpha and beta corticomotor coherence play distinct roles in visuomotor control

**Figure 3.**
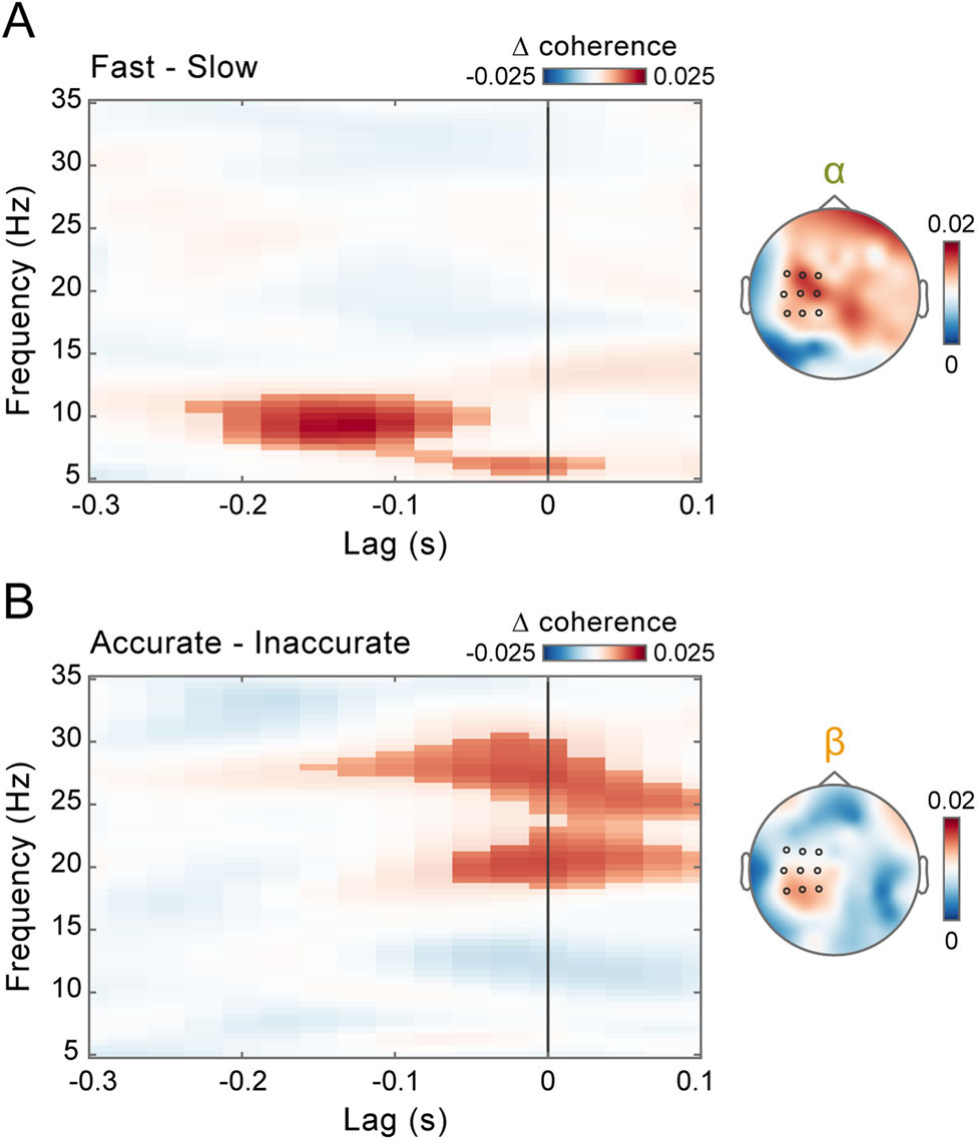
Alpha and beta corticomotor coherence play distinct roles in visuomotor control. ***A***, Lag- and frequency-resolved difference in corticomotor coherence between fast and slow trials (based on a median split), averaged over the selected electrodes (highlighted in black in the topographic map) and the time interval immediately preceding perturbation onset (−0.5 to −0.25 s). The highlighted area indicates the lag (−0.225 to +0.025 s) and frequency (5.5 to 11.5 Hz) intervals belonging to the cluster that survived two-tailed cluster–based permutation statistics for the fast–slow contrast [corrected for multiple comparisons across frequencies (5–35 Hz) and lags (−0.3 to +0.1 s)]. The topographic map shows the fast–slow difference in coherence, averaged over frequencies between 8 and 13 Hz (alpha) and lags between −0.175 and −0.125 s. ***B***, Same as in ***A***, but for the difference between accurate and inaccurate trials. The highlighted area indicates the lag (−0.15 to +0.1 s) and frequency (18.5–30.5 Hz) intervals belonging to the cluster that survived two-tailed cluster–based permutation statistics for the accurate–inaccurate contrast (corrected for multiple comparisons across frequencies (5–35 Hz) and lags (−0.3 to +0.1 s)]. The topographic map shows the accurate–inaccurate difference in coherence, averaged over frequencies between 18 and 30 Hz (beta) and lags between −0.025 and +0.025 s.

As shown, during continuous visuomotor tracking, cortical activities are phase-coupled to the motor output with both spectrally and temporally selective patterns. We tested whether this coupling is functionally relevant for visuomotor control—that is, whether fluctuations in corticomotor coherence are associated with differences in the ability to counteract an unpredictable visual perturbation (i.e., the target jump). To this end, we examined corticomotor coherence during the time interval immediately preceding the target jump (from −0.5 to −0.25 s) for trials with fast versus slow and accurate versus inaccurate responses (based on median splits). Figure 3*A* shows that coherence in the alpha band is stronger for fast trials than for slow trials (cluster *p* = 0.0112; cluster-based permutation test). This modulation peaks at 9 Hz and at a negative lag of −0.125 s, consistent with the lag-tuning profile of alpha coherence. In contrast, when trials were split based on response error, coherence in the beta band around zero lag was higher for accurate responses than for inaccurate ones (Fig. 3*B*; cluster *p* < 0.001).

**Figure 4.**
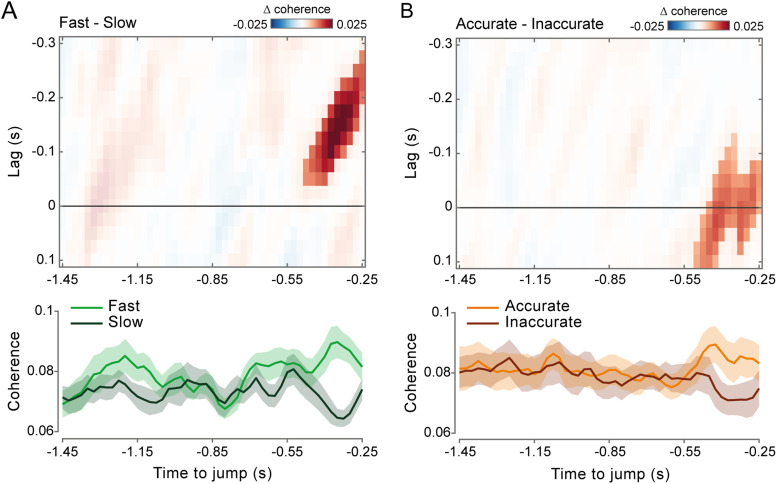
Coherence modulations increase in proximity to the visuomotor perturbation. ***A***, Top, Lag- and time-resolved difference in corticomotor coherence between fast and slow trials (based on a median split), averaged over frequencies between 8 and 13 Hz (alpha) and across the selected electrodes (same as in Figs. 2*D*, 3). The highlighted area indicates the lag (−0.275 to −0.05 s) and time (−0.475 to −0.25 s) intervals belonging to the cluster that survived two-tailed cluster–based permutation statistics for the fast–slow contrast (corrected for multiple comparisons across lags (−0.3 to +0.1 s) and time (−1.45 to −0.25 s)]. Bottom, Time course of corticomotor coherence relative to the target jump, averaged over frequencies between 8 and 13 Hz (alpha) and lags between −0.175 and −0.125 s for fast (light green) and slow (dark green) trials. Shaded areas represent ±1 SEM. ***B***, Top, Same as in ***A*** (top) but showing results for the difference between accurate and inaccurate trials (based on a median split), averaged over frequencies between 18 and 30 Hz (beta). The highlighted area indicates the lag (−0.125 to 0.1 s) and time (−0.5 to −0.25 s) intervals belonging to the cluster that survived two-tailed cluster–based permutation statistics for the accurate–inaccurate contrast [corrected for multiple comparisons across lags (−0.3 to +0.1 s) and time (−1.45 to −0.25 s)]. Bottom, Same as in ***A*** (bottom) but showing results averaged for frequencies between 18 and 30 Hz (beta) and lags between −0.025 and +0.025 s for accurate (orange) and inaccurate (brown) trials.

Next, we examined the modulation of alpha (8–13 Hz) and beta (18–30 Hz) coherence over time. If alpha and beta coherence are functionally relevant for performance, their impact on RT and accuracy should be most pronounced near the time of the target jump. The observed results are consistent with this prediction. The contrasts between fast versus slow (cluster *p* = 0.0096; Fig. 4*A*) and accurate versus inaccurate (cluster *p* = 0.0019; Fig. 4*B*) responses for alpha and beta coherence, respectively, exhibit very similar temporal profiles—both peaking within the 0.5 s preceding the visual perturbation and thus the ensuing motor response.

**Figure 5.**
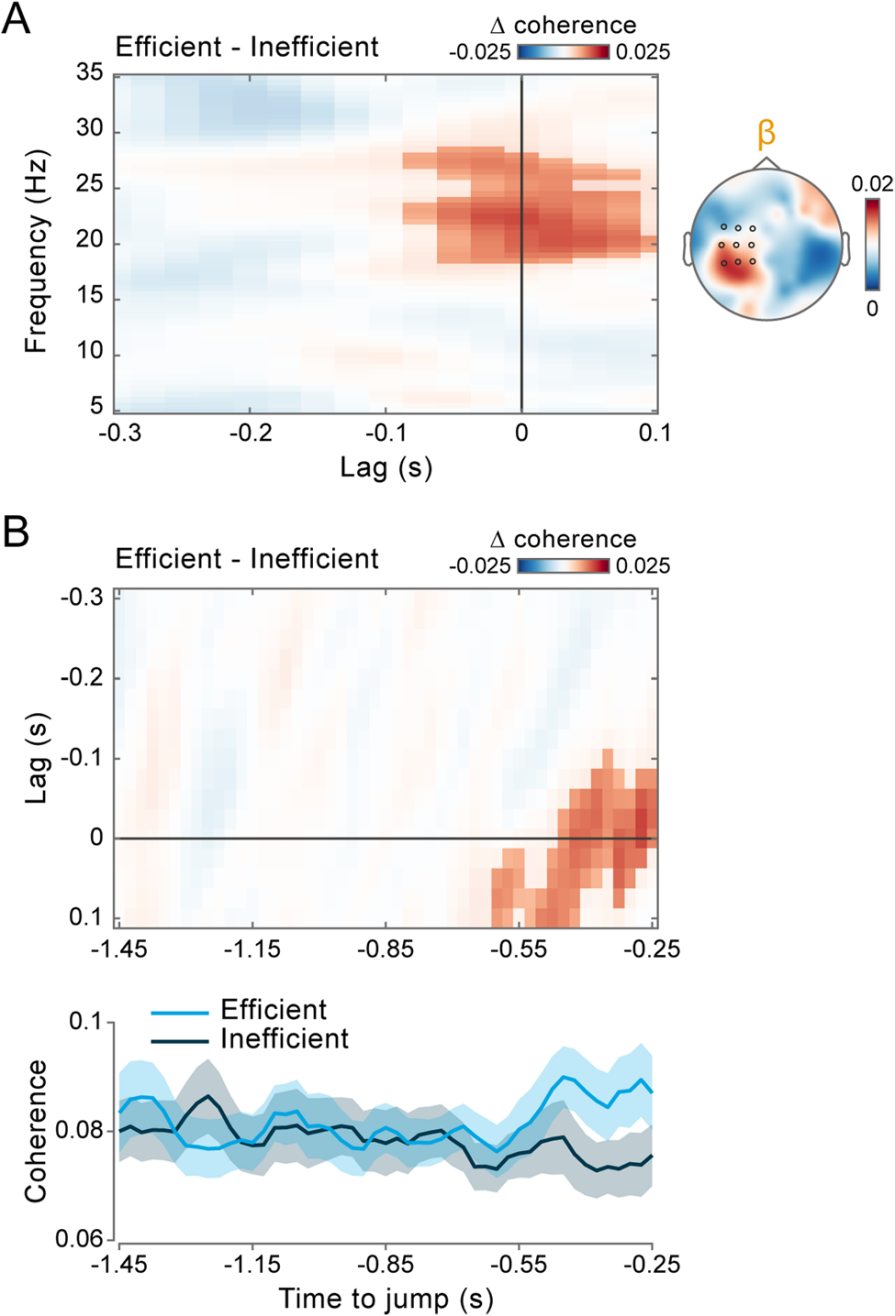
Beta-band coherence supports reward-driven behavior. ***A***, Same analysis as in Figure 3, now showing the difference in coherence between efficient and inefficient trials (classified via a median split based on the efficiency score; Eq. 3). The highlighted area indicates the lag (−0.075 to +0.1 s) and frequency (18.5 to 28.5 Hz) intervals belonging to the cluster that survived two-tailed cluster–based permutation statistics [corrected for multiple comparisons across frequencies (5–35 Hz) and lags (−0.3 to +0.1 s)]. The topographic map shows the efficient–inefficient difference in coherence, averaged over frequencies between 18 and 30 Hz (beta) and lags between −0.025 and +0.025 s. ***B***, Same analysis as in Figure 4, now showing the difference in beta coherence (18–30 Hz) between efficient and inefficient trials. The highlighted area in the top panel indicates the lag (−0.1 to +0.1 s) and time (−0.6 to −0.25 s) intervals belonging to the cluster that survived two-tailed cluster–based permutation statistics (corrected for multiple comparisons across lags (−0.3 to +0.1 s) and time (−1.45 to −0.25 s)]. The bottom panel shows results averaged for frequencies between 18 and 30 Hz (beta) and lags between −0.025 and +0.025 s for efficient (light blue) and inefficient (dark blue) trials.

Alpha and beta coherence appear to be functionally dissociated, predicting the readiness and accuracy of reactive behavior, respectively. This frequency-specific modulation persists independently of the positive association between the two performance metrics (see above; Fig. 1*F*,*G*), as confirmed using a data stratification approach (Fig. S4). To further clarify the functional role of alpha and beta coherence, we computed a performance efficiency score by multiplying on a trial-by-trial basis RTs and response errors, each normalized by their respective performance criteria (i.e., the variable criterion time and the fixed criterion error; see Materials and Methods). The efficiency score quantifies the extent to which performance was steered toward reward attainment. Using a similar median-split analysis, we found that only beta-band coherence was significantly higher in more efficient trials compared with less efficient ones (cluster *p* = 0.0016; Fig. 5*A*), with this modulation again strengthening at time points proximal to the target jump (cluster *p* = 0.0012; Fig. 5*B*).

Altogether, these results suggest that alpha coherence may reflect absolute reactivity, regardless of task demands, while beta coherence may support more strategic behavior aimed at meeting reward contingencies.

### Corticomotor phase relationship predicts performance on a single-trial basis

**Figure 6.**
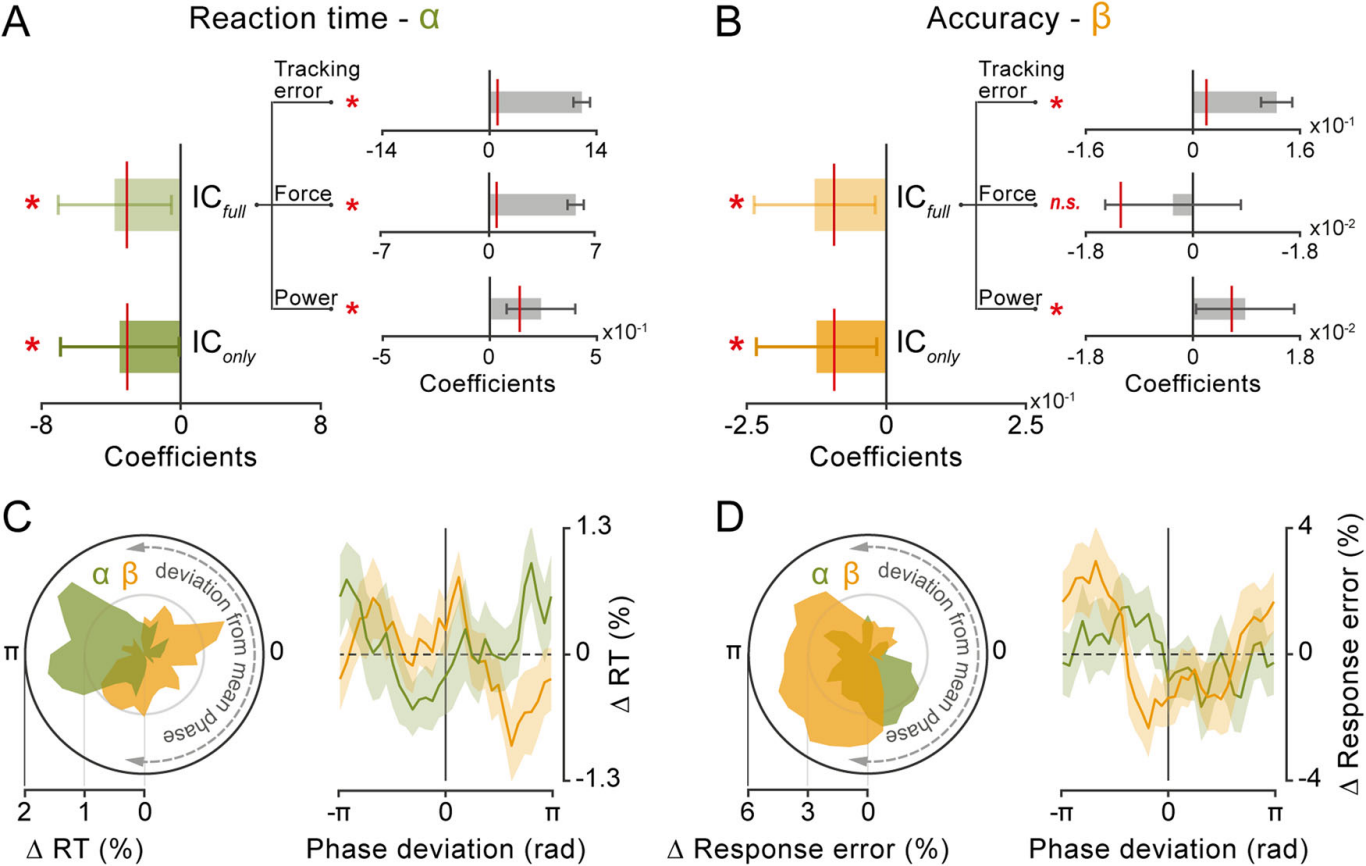
The corticomotor phase relationship predicts performance on a single-trial basis. ***A***, Fixed-effect beta coefficients relative to alpha IC, tracking error, force, and alpha power derived from LME models fitted on RTs. IC and power are averaged over frequencies between 8 and 13 Hz, lags between −0.175 and −0.125 s, and time points between −0.35 and −0.3 s. IC_only_ refers to the IC coefficient derived from the LME model that includes only IC as a predictor, while IC_full_ refers to the IC coefficient derived from the LME model that includes all predictors (i.e., error, force, and power). Error bars indicate the 95% confidence intervals for the fixed-effect coefficients. The red line indicates the 95% threshold based on surrogate data obtained by running the LME model analyses after shuffling the response variable (i.e., RT; 10,000 iterations). ***B***, Same as in ***A*** but for LME models fitted on response error. IC and power are averaged over frequencies between 18 and 30 Hz, lags between −0.025 and +0.025 s, and time points between −0.35 and −0.3 s. ***C***, Left, Polar plot of the distribution of RTs (pooled across all participants) as a function of the deviation from the mean corticomotor alpha (green) and beta (yellow) phase relationship (evaluated at the same time points and band-specific lags as used in the LME model analysis; bin size, 90°; step, 11.25°; bin values expressed as percentage change from the minimum value). Right, Group average of RTs binned as a function of the deviation from the mean corticomotor alpha (green) and beta (yellow) phase relationship (binning parameters as reported above; bin values expressed as percentage change from the mean value). Shaded areas represent ±1 SEM. ***D***, Same as in ***C*** but relative to the distribution of response errors as a function of the deviation from the mean corticomotor phase relationship in both frequency bands.

To investigate whether good reactivity and accuracy relied, respectively, on alpha and beta corticomotor synchronization at an optimal phase relationship, we adopted an approach similar to that used by Rohenkohl et al. (2018) to examine interareal synchronization in monkey data. We derived a point-by-point estimate of the phase-coupling strength between cortical and force signals, referred to as “instantaneous coupling” (IC). This was computed by calculating the deviation between the phase relationship observed at each time point and the “preferred” phase relationship, defined as the mean phase relationship between the signals across time and trials. In other words, the IC quantifies the degree to which the phase relationship between two signals deviates from their mean phase relationship. We estimated IC in the alpha (8–13 Hz) and beta (18–30 Hz) bands, averaging the values over a time window (−0.35 to −0.3 s) and a lag window (alpha, −0.175 to −0.125 s; beta, −0.025 to +0.025 s) centered on the coherence modulation observed in the median-split analysis. We then fitted RT/response error (dependent variables) using separate LME models, with alpha/beta IC as a predictor (fixed-effect factor). As expected, alpha IC significantly predicted RT (*t*_(9378)_ = −2.0174; *p* = 0.0437; standardized beta coefficient = −0.0194; Fig. 6*A*), while beta IC significantly predicted response error (*t*_(9378)_ = −2.3162; *p* = 0.0206; standardized beta coefficient = −0.0233; Fig. 6*B*). The negative sign of the beta coefficients indicates that both shorter RTs and smaller response errors are associated with a cortico-force phase relationship closer to the “optimal” phase in the relevant alpha/beta frequency band. This is further highlighted by binning single-trial RTs and response errors based on the deviation from the mean cortico-force phase relationship in the alpha and beta bands (Fig. 6*C,D*). Both performance metrics exhibit a minimum—indicating better performance—when the phase relationship is close to the mean phase (phase deviation near zero). Performance worsens as the phase deviates toward the opposite relationship (phase deviation near 180°), but this pattern is evident only in the relevant frequency band (i.e., alpha for RT and beta for response error). In contrast, when the nonrelevant frequency band is considered, performance appears relatively uniform across phase values, with no consistent minimum near zero phase.

### Ongoing performance and oscillatory power do not explain the predictive value of coherence

As shown, ongoing alpha and beta corticomotor phase dynamics predict the behavioral outcome. However, two main potential confounding factors may underlie the observed coherence–behavior relationship. One factor is that prejump performance—specifically tracking error and force exerted—is itself predictive of the participants' capacity to react to the visuomotor perturbation (Fig. 1*B–D*). As such, these performance variables may be the actual source of the observed prejump coherence modulation. To control for this confound, we implemented a data stratification procedure. This method ensures that the distributions of relevant control variables—in this case, prejump tracking error and force—are closely matched across trial subsets, in this case, defined by median splits on postjump performance (RT or response error). After stratifying the data separately on tracking error and force, statistical tests on coherence confirmed the original pattern of results: fast trials exhibited increased prejump alpha coherence compared with slow trials (stratified on tracking error, cluster *p* = 0.0061; stratified on force, cluster *p* = 0.009; one-tailed cluster-based permutation test), while accurate trials showed increased beta coherence relative to inaccurate trials (stratified on tracking error, cluster *p* = 0.0013; stratified on force, cluster *p* < 0.001; see Materials and Methods for details).

A second potential confound involves prejump differences in oscillatory power, which may bias coherence estimates. Although power did not differ significantly with response error, fast reactions were preceded by lower power over an extended frequency range (5–35 Hz) compared with slow reactions (Fig. S5). The power modulations displayed distinct topographies (parieto-occipital) and opposite directional patterns (fast < slow) relative to the coherence effects (fast > slow), ruling out a direct contribution of the same oscillatory activity to both effects. Nonetheless, an indirect influence on coherence cannot be excluded. Functionally unrelated yet spectrally overlapping signals—such as occipital alpha—may obscure the activity truly phase-coupled to motor output. Elevated power in these irrelevant sources could impair phase estimation, thereby spuriously reducing coherence values. Therefore, we repeated the stratification procedure, this time controlling for prejump alpha/beta oscillatory power (estimated for the same electrodes and lags as coherence). The results remained virtually unchanged: stronger alpha coherence preceded faster trials (cluster *p* < 0.001; one-tailed cluster–based permutation test) and stronger beta coherence preceded more accurate trials (cluster *p* = 0.0104).

Finally, we exploited the single-trial analysis framework as a robust method to control for any shared explanatory variance between relevant and confounding factors. Specifically, we conducted a regression analysis similar to the one described above, incorporating prejump alpha/beta power, tracking error, and force as additional predictors in each LME model, alongside alpha/beta IC. All three additional factors significantly predicted RT: alpha power (*t*_(9375)_ = 2.9216; *p* = 0.0035; standardized beta = 0.03), tracking error (*t*_(9375)_ = 21.66; *p* < 0.0001; standardized beta = 0.2054), and force (*t*_(9375)_ = 21.07; *p* < 0.0001; standardized beta = 0.1932; Fig. 6*A*). For response accuracy, the observed results were similar, with the exception of force, which showed no significant explanatory power: beta power (*t*_(9375)_ = 2.0902; *p* = 0.0366; standardized beta = 0.0281), tracking error (*t*_(9375)_ = 10.54; *p* < 0.0001; standardized beta = 0.1085), and force (*t*_(9375)_ = −0.5726; *p* = 0.5669; standardized beta = −0.0057; Fig. 6*B*). Most importantly, both alpha IC (*t*_(9375)_ = −2.2805; *p* = 0.0226; standardized beta = −0.0209; Fig. 6*A*) and beta IC (*t*_(9375)_ = −2.2724; *p* = 0.0231; standardized beta = −0.0228; Fig. 6*B*) retained statistically significant explanatory power with comparable effect sizes, demonstrating their independent predictive value.

Taken together, these control analyses strongly suggest that the observed effects of ongoing alpha/beta corticomotor phase on response time/error are not mediated by fluctuations in oscillatory power or baseline behavioral performance and thus are unlikely to reflect unspecific factors such as attention.

## Discussion

Speed and accuracy are key components of sensorimotor control and decision-making. A pivotal question in neuroscience is how sensory information is integrated with the sensorimotor state to generate motor commands for swift and precise actions. In this study, we show that temporally and spectrally selective patterns in corticomotor phase dynamics distinctly favor readiness and accuracy when countering unpredictable visuomotor perturbations. Specifically, stronger alpha-band coherence predicts faster responses, whereas stronger beta-band coherence predicts greater accuracy. Both effects are most pronounced in the final few hundred milliseconds before the perturbation. Single-trial analyses further reveal that performance deteriorates—evidenced by increased response times and errors—as the corticomotor phase relationship deviates from the “preferred” or mean phase. Unlike most previous studies (Androulidakis et al., 2006; Kristeva et al., 2007; Van Wijk et al., 2009; Echeverria-Altuna et al., 2022; Mongold et al., 2022), coherence effects in our data are not confounded by power, highlighting a mechanistic and functional distinction between corticomotor phase dynamics and local amplitude dynamics. Moreover, coherence influences reactive performance independently of ongoing performance—measured via tracking error and force output—excluding the role of nonspecific factors such as attention or net force. Overall, our findings suggest that the interplay between alpha and beta corticomotor coherence reflects neural dynamics within distributed sensorimotor networks that regulate the effective transformation of sensory inputs into motor outputs on a moment-to-moment basis.

Since its discovery (Conway et al., 1995), phase coherence between motor output and cortical activity has been widely documented (Baker et al., 1997; Salenius et al., 1997; Kilner et al., 1999; Schoffelen et al., 2005; Schoffelen et al., 2011; Bourguignon et al., 2017; Mongold et al., 2022), though its role in motor control remains elusive. Coherence is generally stronger in the beta band, which has drawn most attention. Beta-band coherence appears during sustained motor output, decreases with movement, and returns when stability is restored (Baker et al., 1997; Baker et al., 1999; Kilner et al., 1999), paralleling beta-band amplitude dynamics in sensorimotor areas (Pfurtscheller, 1981; Kilavik et al., 2013). Elevated beta power and coherence have also been linked to enhanced responses to somatosensory stimuli (Lalo et al., 2007) and mechanical perturbations (Gilbertson et al., 2005) and improved compensation for visuoproprioceptive mismatches (Androulidakis et al., 2006), suggesting a role in proprioceptive-motor feedback loops that support stability against perturbations (MacKay, 1997; Androulidakis et al., 2007; Baker, 2007). Complementing this evidence, beta activity has been associated with motor inhibition, reduced adaptability, and diminished vigor (Androulidakis et al., 2007; Pogosyan et al., 2009), supporting a prevailing akinetic interpretation (Jenkinson and Brown, 2011), later subsumed under the hypothesis that beta promotes maintenance of the status quo (Engel and Fries, 2010). This picture has since been enriched by novel analytical approaches highlighting the spectral heterogeneity and transient, burst-like nature of beta activity, showing that single-trial burst dynamics closely relate to sensorimotor behavior (Sherman et al., 2016; Van Ede et al., 2018; Little et al., 2019; Diesburg et al., 2021; Rassi et al., 2023; West et al., 2023; Zich et al., 2023). Notably, brief brain bursts are accompanied by muscle bursts and transient increases in corticomuscular coherence, even during sustained isometric contraction (Echeverria-Altuna et al., 2022; but see Mongold et al., 2022). These findings suggest corticomotor coherence may not stem from sustained phase coupling but from coincident—or time-lagged—bursts in brain and peripheral activity (see also Seedat et al., 2020). However, such a mechanism would also imply corresponding power modulations.

Although our analyses captured relatively fast-changing dynamics using data windows consistent with burst duration (0.3 s; Seedat et al., 2020), they were not aimed at detecting single-trial beta bursts. Thus, our results cannot speak to whether beta activity is sustained or transient. Nonetheless, they support at least a partial dissociation between phase coupling and local amplitude dynamics, which challenges the view that corticomotor coherence arises solely from synchronous beta bursts.

Beyond this mechanistic interpretation—which requires support from targeted analyses—our findings also advance the functional understanding of sensorimotor beta. At first glance, they may appear to challenge the traditional akinetic/inhibitory view, as higher beta coherence in our data is linked to more accurate and more efficient—not suppressed—motor responses. To achieve task goals and obtain rewards, participants had to fine-tune their applied force and minimize spatial error within an individually adjusted deadline. In other words, rather than merely rushing, they maximized rewards by striking an optimal balance between accuracy and a relatively flexible speed constraint. This trade-off is more directly captured by the efficiency score, which confirms that higher beta coherence precedes more efficient, reward-oriented behavior. Taken together, the present findings suggest that beta corticomotor phase dynamics may serve as a neural signature of a cautious control strategy, in which overly hasty actions are withheld to maximize the likelihood of reward. This functional account aligns with empirical and theoretical work linking beta activity to reward processing and functional inhibition in support of goal-directed behavior across motor and cognitive domains (Lundqvist et al., 2024).

Conversely, our findings reveal a distinct and complementary role for alpha coherence, which is associated with rapid motor activation in response to visual perturbations.

While beta corticomotor coherence is well documented, evidence for alpha-band coherence is more limited (Raethjen et al., 2002; Williams et al., 2009; Mehta et al., 2014; Piitulainen et al., 2015; Tomassini et al., 2020). The very origin of alpha fluctuations in motor output—commonly termed physiological tremor—remains debated (McAuley and Marsden, 2000). Some emphasize the contribution of cortical (Marsden et al., 2001; Bye and Neilson, 2010), spinal (Christakos et al., 2006), and cerebello–thalamo–cortical loop (Gross et al., 2002) activity, while others propose a peripheral origin, attributing it primarily to biomechanical factors (Lakie et al., 2015). Here, we show that motor alpha, measured from force output, is phase-coherent with cortical alpha. Using lagged coherence analysis, we replicate prior findings (Tomassini et al., 2020), showing that cortical alpha activity precedes motor alpha by ∼200 ms—strongly supporting a neurogenic component of tremor (see Tomassini et al., 2020, for detailed discussion).

Our earlier work also uncovered a link between alpha corticomotor coherence and visual perception: stronger coherence predicted increased detection of near-threshold stimuli. This suggests enhanced sensitivity or a more liberal decision criterion, though hit rates alone cannot distinguish between these two possibilities. Notably, this effect emerged even though the visual stimuli were unrelated to motor output and irrelevant for control—i.e., without any task-mediated coupling between visual and motor processing (Tomassini et al., 2015; Tomassini and D’Ausilio, 2018). We hypothesized that the behavioral relevance of alpha coherence would become clearer under task conditions requiring visuomotor coupling. The present study provides such evidence: during continuous visual-based motor control, alpha coherence speeds up visuomotor transformations, leading to faster responses to unpredictable perturbations.

We suggest that these findings—from both the current and prior study (Tomassini et al., 2020)—reflect ongoing modulations of neuronal excitability and gain, which transiently render visuomotor circuits more excitable and reactive. Some considerations support this view.

RTs are influenced by various neural processes, including local activity and interareal connectivity across frequencies and brain regions (Zhang et al., 2008; Parto-Dezfouli et al., 2023; Rassi et al., 2023). Since RT reflects multiple time-consuming processes—sensory encoding, decision-making, motor preparation, and execution—the interpretation of any RT effect depends on task specifics. Unlike simple RT tasks (Schoffelen et al., 2005), ours incorporated an accuracy component, requiring fast yet finely tuned responses based on dynamically updated sensorimotor evidence, making motor control clearly integrated with decision-making (Gallivan et al., 2018; Thura et al., 2022). A widely reported finding in decision-making research is that speed-accuracy trade-offs are regulated primarily by changes in baseline activity (before evidence accumulation) and gain (during evidence processing) rather than shifts in decision threshold (Reppert et al., 2023). When speed is prioritized over accuracy, baseline activity and gain increase across a broad sensorimotor network (Forstmann et al., 2008; Thura and Cisek, 2016; Reppert et al., 2023), including visual neurons (Heitz and Schall, 2012).

Parallel work grounded in signal detection theory demonstrates similar baseline effects in detection tasks (Van Vugt et al., 2018; Samaha et al., 2020). Lower alpha power—a well-established marker of neural excitability (Haegens et al., 2011; Iemi et al., 2022)—has been associated with increased hit and false alarm rates and shorter RTs (Zhang et al., 2008), without affecting perceptual sensitivity or accuracy (Limbach and Corballis, 2016; Iemi et al., 2017; Kloosterman et al., 2019). This points to an effect on decision criterion, though evidence is mixed (Barne et al., 2020; Zhou et al., 2021). Interestingly, a recent study distinguishes two alpha sources with separate functional roles: visual alpha modulates perceptual sensitivity, while sensorimotor alpha modulates decision criterion (Zhou et al., 2025). Our results align well with this evidence—alpha corticomotor coherence is associated with both increased hit rates (Tomassini et al., 2020) and faster RTs, suggesting a shared mechanism acting at the decisional stage of visuomotor processing.

## Conclusion

We show that corticomotor coherence within the beta and alpha bands contributes to the fine-tuning of online visuomotor control. Motor output retains important information that could be utilized to track the internal dynamics of sensorimotor loops involved in goal-directed behavior, potentially reflecting either a more cautious/conservative (beta) or a more hasty/liberal (alpha) control policy. The present study opens new avenues for future research into the neural mechanisms underlying the flexible balance of two core components of effective behavior—accuracy and speed—with potential implications beyond sensorimotor control to broader domains, including perceptual and cognitive decision-making.

## Data Availability

The raw and preprocessed data, together with the code used to conduct the primary analyses, are available at http://neurolab.unife.it/JN/CMS.zip**.**
